# Severe Diarrhea in a 4-Month-Old Baby Girl with Acute Gastroenteritis: A Case Report and Review of the Literature

**DOI:** 10.1155/2012/920375

**Published:** 2012-03-25

**Authors:** Ionela Loredana Guzganu

**Affiliations:** Department of Pediatrics, Centre Hospitalier de Jolimont-Lobbes, Site Lobbes, Rue Ferrer, 159–7100 Haine-Saint-Paul, Belgium

## Abstract

A 4.5-month-old baby girl presented to hospital with a 2-day history of watery diarrhea and fever. Rehydration and electrolytic balance were restored with intravenous fluid therapy followed by oral rehydration solution but diarrhea did not improve by the fourth day of hospitalization despite treatment with a probiotic. The patient was next treated with gelatin tannate, a medical device recently marketed in Europe to control and reduce the symptoms of diarrhea in infants, children, and adults. The child's diarrhea improved considerably within the first twelve hours and resolved completely within three days. Gelatin tannate might be considered as a useful treatment complementary to oral rehydration solution for the treatment of diarrhea in infants with rotavirus gastroenteritis.

## 1. Introduction

Rotavirus is the leading cause of acute gastroenteritis in infants and children in Europe [1]. Despite improvement of public health across Europe and availability of a vaccine against rotavirus licensed in several countries worldwide, the incidence of intestinal infection remains high and thus an important clinical problem with an equally substantial socioeconomic burden, although mortality has fallen sharply in recent decades. The burden of rotavirus in 2006 for Belgium (prevaccination) was 2.5-fold higher than the median rate estimated for other European countries (3 per 1000, range 0.3–11.9 per 1000) but showed a similar age distribution to neighboring countries [2–4]. The incidence of diarrhea ranges from 0.5 to 1.9 episodes per child per year in children younger than 3 years old in Europe [1]. Although a mild disease in most European countries, acute gastroenteritis is associated with a high number of hospital admissions and high socioeconomic costs. Rotavirus is the most severe enteric pathogen of diarrhea in children. Fever and frequency of vomiting more than twice per day are common symptoms of rotavirus infection, which is considered the main cause of severe dehydrating diarrhea. These signs are frequent in children hospitalized for diarrhea.

The author presents a case of gastroenteritis caused by rotavirus in an otherwise healthy but slightly underweight infant presenting with a 2-day history of severe diarrhea and dehydration.

## 2. Case Presentation

A 4.5-month-old female presented to hospital with a 2-day history of watery diarrhea and fever developed in the 12 hours before admission. The infant was reported to have had 10 watery stools over the previous 24 hours, during which she became quite unsettled, crying a lot, whilst drinking half her usual amount of liquids. There was no history of vomiting.

Her medical history revealed delivery at term via emergency cesarean section due to severe fetal distress, with a birth weight of 2910 g. Her Apgar score was 0-1 at one and five minutes, respectively, after delivery requiring tracheal intubation and transfer into neonatal intensive care unit. She was put into induced coma and active hypothermia for 72 hours. The infant was extubated on the fourth day of life, followed by reestablishment of enteral nutrition by day 5. She was fed exclusively on formula milk from birth. An MRI performed on day 5 showed signs of severe hypoxic-ischemic encephalopathy. Followup after discharge was very irregular and the infant did not receive any vaccines up until the current hospitalization episode. Her weight curve was a reason for concern; indeed, her weight two weeks before the current admission to hospital was 4390 g.

On the day of presentation, physical examination revealed an alert but irritable and ill-appearing infant with a temperature of 39.9°C, heart rate between 170 and 190 beats/min, respiratory rate between 40 and 80 breaths/min, blood pressure of 102/55 mmHg, and oxygen saturation by pulse oximetry of 100%. The child's weight at admission into hospital was 3990 g, meaning she lost 10% of the previous reported weight. The skin was pale grey, tenting skin turgor, dry lips and dry buccal mucosa, normal looking eyes but reduced tears, soft fontanele, and capillary refill time of 3 seconds. The urine output was also decreased. Heart and lung examination were normal except for tachycardia; the abdomen was swollen and slightly painful on palpation, no hepatosplenomegaly. Abdominal and thorax radiographs were normal. There were no signs of meningeal irritation. Laboratory tests showed hemoglobin 12.6 g/dL, white blood cells 11 970/mm3 (PMN = 7590), platelets 1 085 000/mm3, and C-reactive protein was less than 0.05 mg/dL (Table 1). Routine stool specimen tested positive for rotavirus antigen (Vikia Rota-Adeno, Biomerieux), whilst results for blood and urine culture were negative. Serum electrolytes were significant for a sodium concentration of 146 mEq/L and a bicarbonate level of 8 mEq/L. Blood urea nitrogen was 61 mg/dL.

The patient was admitted to the hospital and intravenous fluid therapy was promptly initiated to correct the infant's tachycardia, polypnea, and low blood pH, by attempting volume repletion with 2 subsequent doses of 0.9% sodium chloride at 20 mL/kg over 60 minutes. A solution containing dextrose 5% plus electrolytes was administered soon after the second dose of normal saline to attain the slow correction of hypernatremia. Diuresis was reestablished within four hours of rehydration therapy. Digestive losses of water and electrolytes were offset by oral rehydration solution (ORS), at an initial dose of 10 mL/kg and thereafter replaced volume per volume depending on the amount of stool losses. Enterol (yeast of *Saccharomyces boulardii*) was initiated as an adjuvant treatment to ORS. Metabolic acidosis was difficult to correct during the first 48 hours because of large liquid losses due to diarrhea. Despite the difficulties experienced to restore the electrolytic balance, the baby showed an outstanding digestive tolerance throughout the entire hospital stay, which allowed continuation of enteral nutrition with lactose-free formula milk (Novalac Diarinova). 

At 24 hours after admission, hyperleukocytosis with neutrophilia and increased C-reactive protein in blood (Table 1) prompted antibiotic treatment with ceftriaxone 100 mg/kg/day, which was suspended three days later due to negative bacterial culture results; stools were not tested for the presence of *Clostridium difficile*. Adjuvant treatment with gelatin tannate was initiated on the third day of hospitalization. Metabolic acidosis improved in the following 48 hours along with a substantial reduction in stool output. The baby was exclusively fed by oral route from the fifth day. The patient's history included also antiplatelet treatment (aspirin 5 mg/kg/day for one month) due to thrombocytosis. 

**Figure 1:**
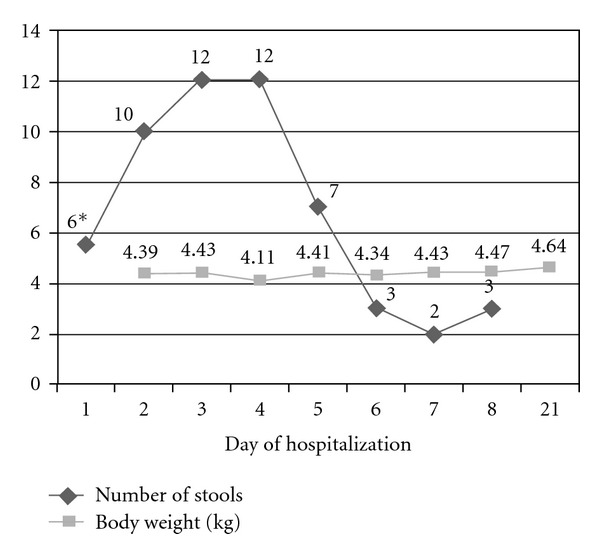
Evolution of both number of daily stools and baby's body weight during hospitalization. Stool count on day one (∗) corresponds to the number of stools since the baby was admitted to hospital from midday on that day.

Gelatin tannate was first administered in the afternoon of the third day of hospitalization, one sachet every 6 hours, five days after the start of diarrhea. The patient presented to hospital with a 2-day history of 10 watery stools a day, followed by a slight increase in the number of liquid stools during the first 4 days of hospitalization (Figure 1). A considerable reduction of the total number of daily stools was registered in the first 24 hours after initiating antidiarrheal treatment with gelatin tannate, with four and three watery stools registered in the first 12 and 24 hours, respectively. The number and volume of stools continued to diminish substantially during the second day of treatment, with the diarrhea practically resolving by the third day of treatment with gelatin tannate (only three stools of almost normal consistency). Gelatin tannate was administered-dissolved in ORS, except for the first dose (dissolved in milk). The progress was favorable, however, the patient was discharged from hospital three days later in order to achieve optimum nutrition and to ensure vaccinations were up to date. Upon discharge from hospital the baby's weight was 4.640 kg.

## 3. Discussion 

Reduced osmolality oral rehydration solution and early refeeding remain central to optimal management of mildly to moderately dehydrated children [1]. Acute gastroenteritis is usually self-limited but a very few antidiarrheal drugs can be considered for more severe cases in infants and children, when symptoms of fever, vomiting and severe diarrhea can seriously aggravate dehydration. Selected probiotics (*Lactobacillus* GG and *Saccharomyces boulardii*) are used to improve intestinal microbial balance and may prove beneficial, although sometimes mild, in reducing the duration of infectious diarrhea mainly of viral etiology [5]. Antibiotics are usually not recommended [1]. 

Fluid and electrolyte replacement are the mainstay of treatment to prevent and correct severe dehydration and acidosis. Oral rehydration therapy is usually sufficient unless the subject is vomiting, and/or losses are very severe. In the present case, IV management with isotonic NaCl followed by the administration of a solution containing dextrose 5% plus electrolytes to restore volume depletion and correct hypernatremia, respectively, were also necessary. 

Strong suspicion of underlying bacterial infection after increase of infection parameters in the 24-hour post-admission laboratory results led to the administration of ceftriaxone discontinued 3 days afterwards due to negative bacterial culture results. 

Given the diarrhea did not improve within the four first days of hospitalization, gelatin tannate was since prescribed. Gelatin tannate has been recently approved in Europe as a medical device to control and reduce the symptoms associated with diarrhea. The use of tannates for the treatment of diarrhea in infants and children has been proven to be effective and safe in previous studies, leading to significant decrease of both the duration of diarrhea and number of watery stools within the first 24 to 48 hours either against placebo [6] or compared to ORS alone [7]. The mechanism of action for gelatin tannate is not entirely clear but it is thought to act locally on the intestinal wall via the formation of a protein-based film thereby protecting the gut from the irritable effect of intestinal secretions responsible for intestinal toxemia. Tannins are well known for their astringent properties, permitting the precipitation of proinflammatory mucoproteins from the intestinal mucus responsible for local inflammation and elimination through the feces [8, 9]. 

The case reported here contributes to the relatively scarce evidence on the efficacy of gelatin tannate in treating diarrhea in infants and children with acute gastroenteritis. More research is needed to elaborate on these findings. 

## Figures and Tables

**Table 1:** Laboratory results.

	Day 1			Day 2		Day 3		Day 4		Day 5	Day 7	Day 21	Normal values
	4 pm	6 pm	11 pm	8 am	3 pm	8 am	4 pm	1 am	8 am	9 am	8 pm	N/A	
WBC/PMN (*10^9^/L)	12/7.6			31.9/24.9	25.8/21.7	13.1/8.1			21.2/8.4	17.2/4	11.2/1.9	8.1/2.6	6–17.5/1.0–8.5
Hb (g/dL)/Ht (%)	12.6/36.4			10.9/31.7	9.1/25.3	9.9/27.4			10/28.5	10.6/30	10.7/29.7	10.8/32.1	11.1–14.1/31–41
Platelets (*10^9^/L)	1085			931	814	620			948	926	940	742	300–750
CRP (mg/dL)	<0.05			1	2	1.3			0.14	0.06	0.05	<0.05	<1
pH	7.21	7.22	7.22	7.23	7.26	7.28	7.26	7.39	7.33	7.45	7.36		7.38–7.46
Bicarbonate/base Excess (mEq/L)	8/−19.3	12/−16	11.4/−16	10/−21.7*	13/−15	11/−18	13/−14	13/−15	15/−12	20.6/−5.4	23/−1.3	23	24–30/(−)3–(+)3
BUN (mg/dL)	61	53		24	18				5		10	17	10–50
Na (mEq/L)	146		145	147	149	156	148	139	141	142	140	136	129–143

BUN: blood urea nitrogen; CRP: C-reactive protein; Hd/Ht: hemoglobin/hematocrit; Na: sodium; N/A: not available; PMN: polymorphonuclear neutrophils; WBC: white blood cells.
